# Digital tools for trial recruitment and retention—plenty of tools but rigorous evaluation is in short supply

**DOI:** 10.1186/s13063-020-04361-8

**Published:** 2020-06-05

**Authors:** Shaun Treweek, Matthias Briel

**Affiliations:** 1 University of Aberdeen, Aberdeen, UK; 2 University of Basel, Basel, Switzerland

Once upon a time checking the weather meant looking out of the window. Now we look at our phones. We bank online, plan holidays online, send our holiday snaps to friends while we are still on holiday using Facebook and WhatsApp and the shop of choice for well, just about anything, is the internet. Can’t find it locally? No worry—go online. Digital is the new black.

The ubiquity of digital is changing randomised trials too. It is a long time since researchers looking for ethical approval have put documents in the post. At the other end of the trial, putting a paper manuscript into an envelope for a journal editor to peruse sounds positively Dickensian. Trial data end up in digital form, and might start out that way. We are all moving, or have moved, to centralised web-based randomisation for most of our trials and the bread-and-butter of multicentre trial communications is email, Zoom, Skype and the like, not water-coolers and coffee machines. The list goes on.

It is no surprise then that trial recruitment and retention also have a digital angle. Recruitment and retention are thorny problems, which makes them top priorities for trial methods work [1]. We need all the help we can get. Digital approaches open up new ways of coming into contact with potential or actual participants and they might improve speed and reduce costs. Two linked articles in *Trials*, one a mapping of published evaluations of digital recruitment and retention tools (Frampton G, Shepherd J, Pickett K, Griffiths G, Wyatt JC: Digital tools for the recruitment and retention of participants in randomised controlled trials: a systematic map, unpublished), the other a survey of what’s happening in the UK in this area [2], provide a useful overview of the current state of play.

First the mapping. Geoff Frampton and colleagues asked ‘What are the types and characteristics of the digital tools that have been evaluated for improving the recruitment and/or retention of people in randomised trials?’ and used a systematic mapping approach to identify and characterise the digital tools they found. They looked at the last 10 years and found quite a lot (105 studies), although the majority (85 studies) were concerned with recruitment alone. To cut to a conclusion, we really do need to talk about retention more; it gets short shrift at present. Awareness-raising was the most commonly evaluated digital recruitment strategy, generally in the form of a website, social media or email. TV and radio feature too, although their star is fading in favour of websites and social media. Email and text messaging crop up most for retention, with both being used to send reminders. Irrespective of whether it is recruitment, retention or both, the majority of evaluations were observational and devilishly tricky to interpret. This was a mapping exercise, not a results synthesis, so the authors do not give recommendations regarding which tools to use, which is perhaps a shame. That said, had they tried they may have failed. In her PhD work [3], Heidi Gardner did a systematic review of non-randomised evaluations of recruitment interventions and found it impossible to say anything about effects for the sort of reasons Frampton and colleagues highlight. It is easy to sign-up to another of the authors’ conclusions: we need more randomised evaluations.

So, that’s the literature; we could do better. The mapping database gives pointers to areas in need of study (e.g. how to recruit and retain ethnic minorities) and it is worth spending some time playing with the spreadsheet—it’s fascinating.

The second study by Amanda Blatch-Jones and colleagues [2] asked staff at UK Clinical Trial Units what digital recruitment and retention tools they use and asked staff at the National Institute for Health Research (a UK trial funder) about the digital tools their funded trials use. In both cases responses were received from about half of those eligible. Websites and social media featured highly for both groups as recruitment and retention tools, again chiefly to raise awareness of trials. Clinical Trial Unit staff mentioned more recruitment tools (41) than retention ones (29), though that is a smaller difference than we might have expected. Nearly half of the recruitment tools focused on identification of potential participants; more than half of the retention tools used SMS or email reminders. The qualitative interviews that were part of this study suggest that trialists, potential participants and members of ethics committees are largely relaxed about the use of digital tools for recruitment and retention as long as security is handled well.

But there are other messages. One is that we have little high quality evidence about the effectiveness or otherwise of these tools, which was also a conclusion from the mapping study. Staff are pretty positive about some of the recruitment tools, less so about the retention ones. But this is not the same as having robust evidence about effects. Another message is that most people think digital tools will be part of a mixed approach to recruitment and retention. In other words, they have a potentially important role but they will not solve all our problems. Many people like to talk to someone about taking part, not just take part.

As with the rest of our lives, we can no longer ignore digital and why should we. There is no justification for dismissing digital tools for recruitment and retention out of hand, but, as the authors of both articles conclude, it would be nice to know that at least some of them actually work. That requires more rigour. How to do that is not rocket science—it is the same approach we already use to answer the research questions in our clinical trials.

## Data Availability

Not applicable.
